# Perceived misdiagnosis of psychiatric conditions in autistic adults

**DOI:** 10.1016/j.eclinm.2024.102586

**Published:** 2024-04-04

**Authors:** Vasiliki Kentrou, Lucy A. Livingston, Rachel Grove, Rosa A. Hoekstra, Sander Begeer

**Affiliations:** a Department of Experimental and Applied Psychology, Faculty of Behavioural and Movement Sciences, Vrije Universiteit Amsterdam, Amsterdam, the Netherlands; b Department of Psychology, Institute of Psychiatry, Psychology and Neuroscience, King's College London, London, United Kingdom; c School of Public Health, Faculty of Health, University of Technology Sydney, Sydney, Australia; d Department of Clinical Developmental Psychology, Faculty of Behavioural and Movement Sciences, Vrije Universiteit Amsterdam, the Netherlands

**Keywords:** Autism spectrum disorder, Autism, Misdiagnosis, Prior diagnoses, Adults

## Abstract

**Background:**

Many autistic people, particularly women, do not receive an autism diagnosis until adulthood, delaying their access to timely support and clinical care. One possible explanation is that autistic traits may initially be misinterpreted as symptoms of other psychiatric conditions, leading some individuals to experience misdiagnosis of other psychiatric conditions prior to their autism diagnosis. However, little is currently known about the frequency and nature of psychiatric misdiagnoses in autistic adults.

**Methods:**

Using data collected in the first half of 2019 from an ongoing longitudinal register of autistic adults in the Netherlands, this study explored the frequency of perceived psychiatric misdiagnoses before receiving an autism diagnosis. Gender differences were also explored. A sample of 1211 autistic adults (52.6% women, mean age 42.3 years), the majority of whom were Dutch and relatively highly educated, was evaluated.

**Findings:**

Results showed that 24.6% (*n* = 298) of participants reported at least one previous psychiatric diagnosis that was perceived as a misdiagnosis. Personality disorders were the most frequent perceived misdiagnoses, followed by anxiety disorders, mood disorders, chronic fatigue syndrome/burnout-related disorders, and attention-deficit/hyperactivity disorder. Autistic women (31.7%) reported perceived misdiagnoses more frequently than men (16.7%). Women were specifically more likely than men to report perceived misdiagnoses of personality disorders, anxiety disorders, and mood disorders. Women also reported prior psychiatric diagnoses more often in general (65.8% versus 34.2% in men). Within the group of individuals with a prior diagnosis, perceived misdiagnoses were equally likely for men and women.

**Interpretation:**

One in four autistic adults, and one in three autistic women, reported at least one psychiatric diagnosis, obtained prior to being diagnosed with autism, that was perceived as a misdiagnosis. Inaccurate diagnoses are linked to long diagnostic pathways and delayed recognition of autism. These findings highlight the need for improved training of mental health practitioners, in order to improve their awareness of the presentation of autism in adulthood and of the complex relationship between autism and co-occurring conditions. The current study constitutes a first step towards showing that autistic adults, and particularly women, may be at greater risk of experiencing misdiagnoses. Future studies based on larger, more representative samples are required, to replicate current findings and provide more reliable estimates of the overall frequency of misdiagnoses as well as the frequency of misdiagnoses for specific psychiatric conditions.

**Funding:**

This study was made possible by funding from the 10.13039/501100001826Netherlands Organisation for Health Research and Development (ZonMW), project number 60-63600-98-834.

**Research in context**

**Evidence before this study**

Before undertaking this study, a number of databases, including PubMed, PsycInfo, EBSCOhost, and Google Scholar, were searched. We aimed to identify any literature reporting on misdiagnoses (and prior or co-occurring diagnoses) of psychiatric conditions in autistic adults. Search strings that were used included (“autism” OR “autism spectrum disorder” OR “asd”) AND (“misdiagn∗” OR “co-occurring” OR “comorbid”). The initial search was performed in June 2019 and the most recent search was performed in September 2022. The search was not limited to English language publications. Additional literature was also identified by inspecting reference lists of relevant articles. Reviewing all relevant articles revealed a small body of qualitative research detailing the experiences of late-diagnosed autistic adults, in which suspected misdiagnosis often emerged as a key theme. The search also revealed a small number of case studies describing misdiagnosis of various psychiatric conditions, such as personality disorders, in autistic adults. However, no large-scale studies providing quantitative estimates of perceived misdiagnoses of different psychiatric conditions in autistic adults were identified. Our literature search also revealed that autistic women could be more likely than men to experience misdiagnoses, although this had not yet been empirically tested.

**Added value of this study**

Ensuring that autistic adults receive timely access to treatment and care has important clinical implications. Nevertheless, little is known about the nature of misdiagnoses in autistic adults, including whether certain psychiatric conditions are more likely than others to be misdiagnosed in autistic adults, or whether any gender-specific effects are present. This study was one of the first to show that approximately a quarter of autistic adults reported being misdiagnosed with at least one psychiatric condition before receiving an autism diagnosis. Personality disorders, mood disorders, and anxiety disorders were most frequently perceived as misdiagnoses. Importantly, women reported misdiagnoses more frequently than men. Women were also more likely than men to report misdiagnoses of personality disorders, anxiety disorders, and/or mood disorders.

**Implications of all the available evidence**

Our findings highlight the importance of screening for autism earlier in adults, especially those presenting with numerous prior or current diagnoses of personality, anxiety, or mood disorders. Clinicians working with adults in psychiatric settings, both men and women, are encouraged to be aware of the nuanced presentations of autistic traits and consider the impact of compensatory and camouflaging strategies on the behavioural presentation of autism. Importantly, clinicians should be aware that autistic men and women are potentially susceptible to different types of misdiagnoses, and that women in particular are more susceptible than men to misdiagnosis of personality disorder. In order to reduce risk of misdiagnosis, mental health assessments in autistic adults should form a key component of clinical care with regular screening, evaluation, and treatment done as part of ongoing support, rather than treating psychiatric conditions in isolation.

## Introduction

Autism is characterised by early-onset differences in social interaction and communication, repetitive and restricted behaviours, and sensory sensitivities.^1^ It is now increasingly recognised that autism might not be identified until adolescence or adulthood,^2^ but obtaining an autism diagnosis later in life remains challenging. Although it is estimated that 70–80% of autistic adults will also meet diagnostic criteria for at least one other psychiatric condition,^3^^,^^4^ a previous study has shown that only half of autistic adults agree with their co-occurring diagnoses.^5^ Similarly, approximately half of adults without a formal autism diagnosis who suspect they may be autistic do not fully agree with their psychiatric diagnoses.^5^ One explanation is that autistic characteristics may be masked by or misinterpreted as symptoms of other psychiatric conditions,^5^, ^6^, ^7^, ^8^, ^9^ thereby delaying the diagnosis of autism.^6^^,^^10^^,^^11^

Co-occurring psychiatric conditions are highly prevalent in autistic adults.^12^, ^13^, ^14^, ^15^ Research examining the prevalence of psychiatric and physical conditions in a large and diverse sample (*N* = 1507) demonstrated that, relative to sex-matched non-autistic controls, autistic adults experienced an elevated risk of most psychiatric conditions, including anxiety, depression, bipolar disorder, obsessive-compulsive disorder, and schizophrenia, as well as a five-fold risk of suicide attempts.^12^ Relative to controls, autistic adults were also at greater risk of nearly all chronic medical conditions, including autoimmune conditions, sleep and gastrointestinal disorders, seizure, obesity, hypertension and diabetes.^12^ Autistic women were diagnosed more often than autistic men with anxiety, bipolar disorder, dementia, depression, schizophrenia, and suicide attempts, and the risk of dementia, psychoses, and schizophrenic disorders was substantially higher in autistic women than men.^12^ A subsequent study using a national sample of autistic older adults (*N* = 4685) similarly showed that, relative to a matched population comparison cohort, the majority of mental and physical health conditions were significantly more prevalent in autistic older adults.^13^ Using representative data from Swedish population registers, Martini et al. (2022) also concluded that, relative to non-autistic same-sex individuals, autistic males and females were both at increased risk of nearly all psychiatric diagnoses as well as psychiatric hospitalization for all disorders. Importantly, this study also explored sex differences in psychiatric diagnoses and psychiatric hospitalization among autistic relative to non-autistic young adults, and found significant sex differences in the cumulative incidence of psychiatric diagnoses, with 77% of autistic females and 62% of autistic males receiving at least one psychiatric diagnosis.^14^

A key factor underlying the risk of misdiagnosis is diagnostic overshadowing, whereby autistic traits are either missed or misattributed to different psychiatric diagnoses,^5^^,^^16^ such as personality disorders, eating disorders, trauma-related disorders, bipolar disorder, psychosis, or schizophrenia.^5^, ^6^, ^7^^,^^17^^,^^18^ Indeed, previous research has shown symptom overlap between autism and numerous psychiatric conditions, including attention deficit/hyperactivity disorder,^19^, ^20^, ^21^ generalised anxiety disorder,^22^ obsessive-compulsive disorder,^22^^,^^23^ social anxiety,^2^ personality disorders,^9^^,^^24^^,^^25^ and eating disorders.^26^^,^^27^ Moreover, living without an established autism diagnosis is linked to negative experiences involving feelings of distress, isolation, anxiety, or confusion, which can serve as catalysts for different psychiatric conditions before autism is diagnosed.^6^^,^^28^, ^29^, ^30^, ^31^ Healthcare professionals may therefore focus on the treatment of more readily observable mental health symptoms, possibly failing to identify when these may potentially be manifestations of underlying autistic symptoms or consequences of living with undiagnosed autism, further reducing the chances of receiving an autism diagnosis.^5^^,^^28^ Lack of awareness surrounding the nuanced presentation of autistic traits in adulthood is also thought to increase the likelihood of diagnostic overshadowing and, ultimately, misdiagnosis; for example, when a client presents with (genuine) anxiety, and the clinician fails to investigate further to detect the underlying autism.^5^^,^^29^^,^^30^^,^^32^ Camouflaging and compensatory strategies, which often characterise individuals receiving a later autism diagnosis in adulthood, may pose an additional barrier to the timely recognition of autism.^2^^,^^33^, ^34^, ^35^ When core autistic difficulties are masked by these strategies (e.g., mimicking neurotypical communication styles^29^), observable autistic traits are less likely to be identified by healthcare professionals or trigger a specialised assessment.^34^^,^^36^^,^^37^ This is particularly problematic when camouflaging and compensatory techniques, which are linked to poorer mental health outcomes, are employed, either consciously or automatically, during diagnostic assessments.^29^^,^^38^

Although the possibility of experiencing a misdiagnosis is present in both men and women, autistic women may be more likely than men to experience a misdiagnosis.^10^^,^^39^^,^^40^ This has been attributed to the ‘female autism phenotype’, a behavioural presentation of autism that is more common in women and may be expressed in ways that differ from male phenotypes. For example, some women may demonstrate milder social communication difficulties and fewer stereotyped or repetitive behaviours,^41^ and are often perceived as having greater social awareness, higher social motivation, and superior cognitive flexibility and emotion recognition skills, relative to men.^42^^,^^43^ In addition, compared with their typically-developing peers, some autistic women may also demonstrate relatively intact neural self-representation and mentalising abilities.^33^ This female-specific cognitive pattern might confer an advantageous ability to analyse and process observations of subtle social cues during interpersonal interactions,^44^ enabling certain women to camouflage autistic characteristics more successfully than men.^45^^,^^46^ However, successful camouflaging may prevent the manifestation of overt functional impairments in social communication,^34^^,^^35^ possibly leading to late diagnoses or misdiagnoses.^2^^,^^37^^,^^39^^,^^40^^,^^47^

Despite increasing research interest into misdiagnosis of psychiatric conditions diagnosed prior to autism, and the important clinical implications of ensuring that autistic people receive timely access to appropriate diagnosis and care, relatively little is known about the nature of perceived misdiagnoses in autistic adults. First, it is unclear which prior psychiatric conditions are most likely to be perceived as misdiagnoses by autistic adults. Second, it is unclear whether certain prior psychiatric conditions are more or less likely to be perceived as misdiagnoses by autistic women relative to men. Based on a sample of 1211 Dutch autistic adults, the current study aimed to quantify the frequency of self-reported perceived misdiagnoses of psychiatric conditions diagnosed before a diagnosis of autism was obtained, and to compare the frequency of self-reported perceived misdiagnoses of prior conditions between men and women.

## Methods

### Participants

#### The Netherlands autism register

Data were obtained from the Netherlands Autism Register (NAR), a longitudinal volunteer register containing data from approximately 3500 autistic individuals. The NAR was established in 2013 by the Dutch Autism Association (NVA) in collaboration with the Vrije Universiteit Amsterdam, and is distributed to new and recurring respondents on an annual basis. It has received ethical approval from the Vaste Commissie Wetenschap en Ethiek (VCWE) of the Vrije Universiteit Amsterdam. It contains information on multiple domains, including general demographics, diagnosis and diagnostic history, autism symptom profile, co-occurring diagnoses, treatment, education, employment, well-being, interpersonal relationships, as well as sensory processing, physical health, special interests, and cognitive functioning. Every year, a link to join the survey is advertised both to members and non-members of the NVA on the association's website (www.autisme.nl), on the NAR website (www.nar.vu.nl), as well as during regular presentations given by researchers working with the NAR. Approximately 1% of the Dutch autistic population is represented in the NAR. Participation is free and voluntary, and each participant receives individual feedback generated from their survey responses.

#### Sample characteristics

In the current study, participants were aged 16 years or older at the time of their first participation and provided written informed consent before participating. All participants had a formal diagnosis of a pervasive developmental disorder according to the Diagnostic and Statistical Manual of Mental Disorders-Fourth Edition (DSM-4^48^), or autism spectrum disorder according to the DSM-5,^1^ made by a qualified clinician unaffiliated with the current study. Questions regarding perceived misdiagnoses were only posed to respondents who took part in the fifth wave of the survey. Therefore, only respondents from wave 5 were included in the current study, resulting in a final sample size of 1211 respondents, of whom 52.6% (*n* = 637) identified as women and 47.4% (*n* = 574) identified as men. Participants' age ranged from 16 to 85 years (*M* = 42.26, *SD* = 15.59). The average age for women was 41.33 years (*SD* = 13.72, range: 16–80), whereas the average age for men was 43.28 years (*SD* = 17.38, range: 16–85). The majority of participants (95.7%, *n* = 1115) were Dutch. Regarding their education, 8.8% (*n* = 87) reported a low level of education, 44.7% (*n* = 444) reported a middle level of education, whereas 46.6% (*n* = 463) reported a high level of education, making the current sample relatively highly educated. The majority of participants received their autism diagnosis in adulthood (*M* = 31.99 years, *SD* = 17.56). Age of autism diagnosis did not differ between men and women (*t* (962) = 0.19, *p* = 0.849, 95% CI of the difference = [−1.91 to 2.32]). However, after controlling for a significant gender difference in current age, whereby men were older than women (*t* (1087) = 2.16, *p* = 0.031, 95% CI = [0.18–3.73], women had a later age of autism diagnosis compared to men (*F* (1,1050) = 40.02, *p* < 0.001). There was no difference in autistic traits between men and women (*t* (960) = 1.09, *p* = 0.273, 95% CI = [−2.09 to 0.59]), even when controlling for participants’ current age (*F* (1,1101) = 3.25, *p* = 0.072; Table 1). Table 1, Table 2 contain additional participant characteristics.

**Table 1:** Sample characteristics.

	Male		Female		Total	
	*n*	%	*n*	%	*n*	%
Ethnicity (*n* = 1165)						
Dutch	532	92.7	605	95	1137	93.9
Indonesian	3	0.5	5	0.8	8	0.7
Surinamese	0	0	4	0.6	4	0.3
Antillean, Aruban	1	0.2	0	0	3	0.2
Moroccan	0	0	1	0.2	1	0.1
Turkish	1	0.2	0	0	1	0.1
Education level (*n* = 994)						
Low	53	11.9	34	6.2	87	8.8
Middle	202	45.5	242	44	444	44.7
High	189	42.6	274	49.8	463	38.2
Highest completed education (*n* = 1028)						
Primary education	9	1.9	14	2.5	23	2.2
Primary special education	23	4.9	8	1.4	31	3.1
Pre-vocational secondary education	35	7.6	37	6.5	72	7
General secondary education	24	5.2	48	8.5	72	7
Pre-university secondary education	34	7.3	38	6.7	72	7
Secondary special education	20	4.3	10	1.8	30	3
Secondary vocational education	100	21.6	114	20.2	214	20.8
Higher vocational education	106	22.9	152	26.9	258	25.1
University (Bachelor/Master/Doctorate)	83	17.9	122	21.6	205	19.9
No fully completed education	10	2.2	7	1.2	17	1.7
Other/unclear	19	4.2	15	2.7	34	3.2
Gross annual income in current household (*n* = 940)						
€1–€10,000	23	5.0	35	7.2	58	6.2
€10,000–€20,000	110	24.1	164	34.0	274	29.1
€20,000–€30.000	66	14.4	67	13.9	133	14.1
€30,000–€40.000	63	13.8	74	15.3	137	14.6
€40,000–€50.000	59	12.9	47	9.7	106	11.3
€50,000–€60.000	38	8.3	35	7.2	73	7.8
€60,000–€70.000	26	5.7	21	4.3	47	5.0
€70.000 or higher	72	15.8	40	8.3	112	11.9
Intellectual disability (*n* = 1207)						
No	510	88.9	605	95	1115	92.1
Yes	44	7.7	24	3.8	68	5.6
Unclear/conflicting information	16	2.8	8	1.3	24	2
Age of autism diagnosis in categories (*n* = 1104)						
<18	159	31.0	115	19.5	274	23.8
18–24	25	4.9	83	14.0	108	9.8
25–34	50	9.7	137	23.2	187	16.9
35–44	106	20.7	128	21.7	234	21.2
45–54	112	21.8	90	15.2	202	18.3
≥55	61	11.9	38	6.4	99	9.0
At least one current co-occurring diagnosis (*n* = 1211)						
Yes	205	35.7	328	51.5	533	44
No	338	58.9	281	44.1	619	51.1
Unsure/unknown	31	5.4	28	4.4	59	4.9

**Table 2:** Participant characteristics, including current age, age of autism diagnosis, total and factor-level scores on the AQ-Short, age when autism was first considered, and time between first consideration of autism and formal autism diagnosis.

	Male		Female		Total	
	*M*	*SD*	*M*	*SD*	*M*	*SD*
Current age, years (*n* = 1211)	43.3	17.4	41.3	13.7	42.3	15.6
Age of autism diagnosis, years (*n* = 1104)	32.1	19.8	31.9	15.4	32.0	17.6
AQ-Short total score (*n* = 1050)	82.9	11.5	83.6	10.5	83.3	10.9
Social skills	21.2	4.1	21.7	3.6	21.5	3.8
Routine	12.1	2.4	12.7	2.2	12.4	2.3
Switching	13.1	2.2	13.2	2.1	13.2	2.2
Imagination	22.9	4.4	22.9	4.5	22.9	4.4
Numbers and patterns	13.7	3.8	13.1	3.9	13.4	3.8
Social behaviour	60.5	8.4	61.7	7.6	61.2	7.9
Age when autism was first considered, years (*n* = 1042)	27.4	20.3	29.0	16.7	28.2	18.5
Time between first consideration and formal autism diagnosis, years (*n* = 1011)	4.2	9.0	3.3	6.9	3.7	8.0

### Measures and procedure

#### Perceived misdiagnoses

**Fig. 1:**
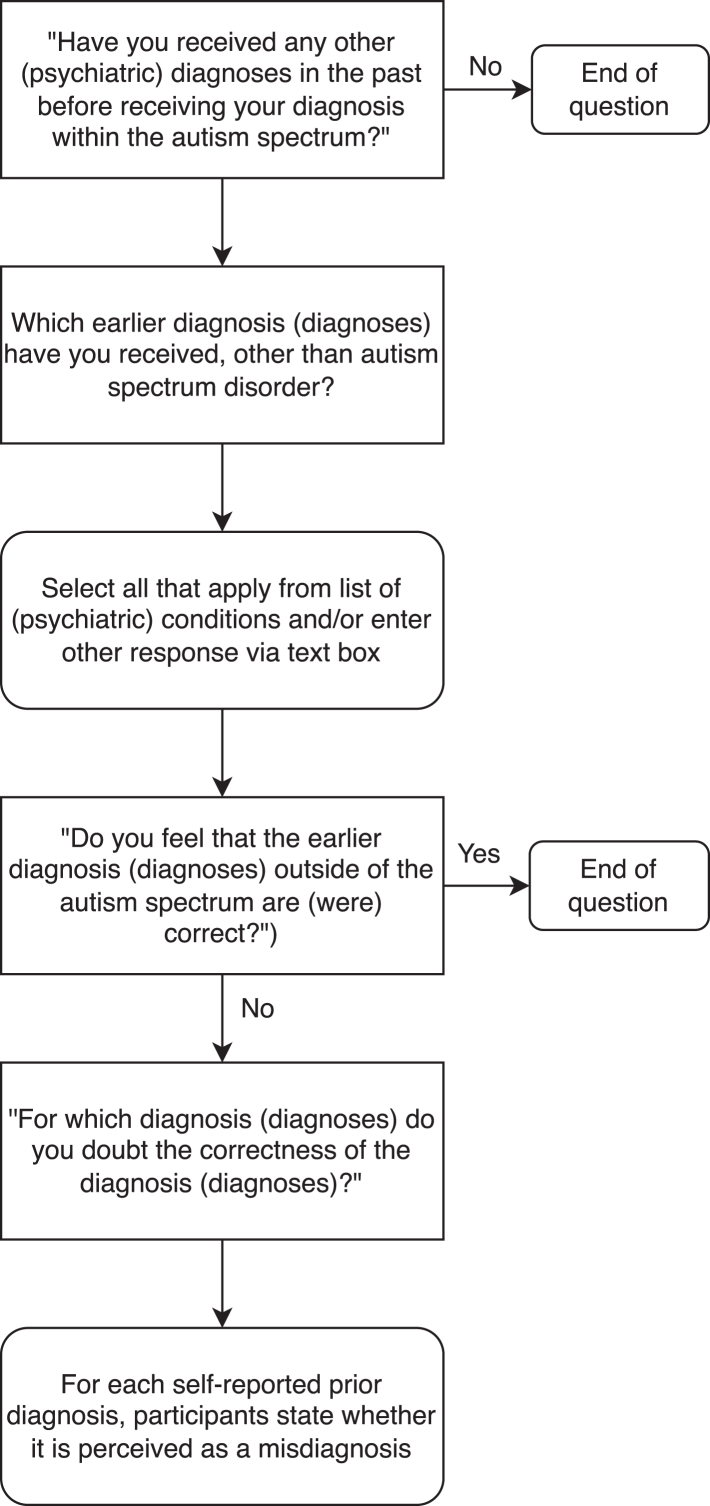
Flowchart of questions regarding prior diagnoses and perceived misdiagnoses.

When entering the register, participants were asked if they had received any additional psychiatric diagnoses in the past, before receiving a diagnosis of autism (“Have you received any other (psychiatric) diagnose(s) in the past before receiving your diagnosis within the autism spectrum?”). This was followed by the question “Which earlier diagnosis (diagnoses) have you received, other than autism spectrum disorder?” A drop-down list of conditions was presented, including attention deficit/hyperactivity disorder (ADHD/ADD), mood disorders (including major depressive disorder, dysthymia, and bipolar disorder), anxiety disorders (including specific phobia, generalised anxiety disorder, and obsessive-compulsive disorder), personality disorders, post-traumatic stress disorder (PTSD) or other trauma-related disorders, chronic fatigue syndrome (CFS) or burnout-related disorders, and fibromyalgia. Multiple selection was allowed, and participants were also given the option to enter the name of a condition not included in the list. As a result, eating disorders, substance use disorder (SUD), and oppositional defiant disorder or conduct disorder (ODD/CD) were also included in the analyses. Participants who reported having one or more prior diagnoses were then asked whether this was perceived as a misdiagnosis (“Do you feel that the earlier diagnosis (diagnoses) outside of the autism spectrum are (were) correct?”). If participants indicated that one or more of their earlier diagnoses were incorrect, they were then asked to select which specific psychiatric conditions they perceived as incorrect (“For which diagnosis (diagnoses) do you question the correctness of the diagnosis (diagnoses)?”). Fig. 1 includes a visual representation of the question flow.

#### Autistic traits

Autistic traits were measured using the Autism-Spectrum Quotient-Short (AQ-Short),^49^ a 28-item self-report measure that includes two subscales assessing social behaviour and an interest in numbers and patterns, thus mapping onto social and non-social features of autism.^49^ Participants respond using a 4-point Likert scale ranging from “1 = definitely agree” to “4 = definitely disagree”, with total scores ranging from 28 to 112. Higher scores indicate a higher endorsement of autistic traits. The measure showed acceptable to good internal consistency in previous studies.^49^ In the current study, internal consistency was good for the total AQ-Short (Cronbach's α = 0.84) and the social behaviour factor (α = 0.84), and acceptable for the numbers/patterns factor (α = 0.78).

### Statistical analyses

All statistical analyses were performed using IBM SPSS Statistics (Version 27). We first explored rates of perceived misdiagnoses of psychiatric conditions diagnosed prior to autism. The association between gender and perceived misdiagnoses was then examined using a chi-square test of independence. Binary logistic regression was used to examine the probability of experiencing a misdiagnosis as a function of gender, while controlling for participants’ current age and other factors. Five separate logistic regression models were tested to examine the association between gender and the probability of being misdiagnosed with: i) personality disorders, ii) mood disorders, iii) anxiety disorders, iv) CFS/burnout-related disorders, and v) ADHD/ADD. The associations between gender and the probability of being misdiagnosed with other conditions were not examined due to the small number of perceived misdiagnoses reported. To account for multiple hypothesis testing, the Bonferroni correction was applied, with an adjusted alpha level of 0.01.

### Role of the funding source

This study was made possible by funding from the Netherlands Organization for Health Research and Development (ZonMW), project number 60-63600-98-834. The funding source was not involved in the study design, data collection, analysis, interpretation, writing of the manuscript, or the decision to submit it for publication.

## Results

### Frequencies of prior diagnoses

First, the proportion of respondents who reported at least one diagnosis for a psychiatric condition diagnosed prior to autism was examined. Out of the total sample of 1211 individuals, 42.9% (*n* = 520) reported having received at least one psychiatric diagnosis prior to receiving an autism diagnosis. Women (53.7%) were more likely than men (31%) to report at least one prior diagnosis, *b* = 0.96, *SE* = 0.14, Wald *χ*^*2*^ = 49.39, *p* < 0.001, *OR* = 2.62 (95% = [2.00–3.43]), controlling for participants' current age, ethnicity, gross annual income in their current household, and highest level of completed education. Individuals with a higher age of autism diagnosis were also more likely to report at least one prior psychiatric diagnosis, *b* = 0.02, *SE* = 0.01, Wald *χ*^*2*^ = 19.17, *p* < 0.001, *OR* = 1.02 (95% = [1.01–1.03]), controlling for participants’ gender, ethnicity, gross annual income in their current household, and highest level of completed education.

### Frequencies of perceived misdiagnoses

Second, the proportion of respondents who reported at least one perceived misdiagnosis for a psychiatric condition diagnosed prior to autism was examined. A quarter of all participants (*n* = 298; 24.6%) reported at least one prior psychiatric condition which they perceived as a misdiagnosis. Women (31.7%) were more likely than men (16.7%) to report at least one perceived misdiagnosis, *b* = 0.79, *SE* = 0.15, Wald *χ*^*2*^ = 26.22, *p* < 0.001, *OR* = 2.19 (95% = [1.62–2.69]), controlling for participants' current age, ethnicity, gross annual income in their current household, and highest level of completed education. Individuals with a higher age of autism diagnosis were also more likely to report at least one perceived misdiagnosis, *b* = 0.01, *SE* = 0.01, Wald *χ*^*2*^ = 8.07, *p* = 0.004, *OR* = 1.01 (95% = [1.00–1.03]), controlling for participants’ gender, ethnicity, gross annual income in their current household, and highest level of completed education. When the analysis was narrowed down only to participants who had reported at least one prior diagnosis (n = 520), neither gender, *b* = 0.14, *SE* = 0.21, Wald *χ*^*2*^ = 0.47, *p* = 0.492, *OR* = 1.01 (95% = [0.77–1.72]), nor age of autism diagnosis was predictive of the probability of reporting at least one perceived misdiagnosis, *b* = 0.00, *SE* = 0.01, Wald *χ*^*2*^ = 0.57, *p* = 0.955, *OR* = 1.00 (95% = [0.99–1.0]).

### Perceived misdiagnoses for specific diagnostic categories

The proportion of self-reported perceived misdiagnoses for specific diagnostic categories was then examined. Out of the total sample, 12.3% (*n* = 149) reported a perceived misdiagnosis of a personality disorder, 6.6% (*n* = 80) an anxiety disorder, and 6.2% (*n* = 75) a mood disorder. Additionally, 4% (*n* = 49) perceived their diagnosis of CFS/burnout-related disorder as a misdiagnosis, while 2.8% (*n* = 34) reported a perceived misdiagnosis of ADHD/ADD. Women were more likely than men to report a perceived misdiagnosis for personality disorders, *b* = 1.05, *SE* = 0.21, Wald *χ*^*2*^ = 24.87, *p* < 0.001, *OR* = 2.87 (95% = [1.89–4.34]), anxiety disorders, *b* = 0.62, *SE* = 0.26, Wald *χ*^*2*^ = 5.62, *p* = 0.018, *OR* = 1.86 (95% = [1.11–3.12]), and mood disorders, *b* = 0.86, *SE* = 0.28, Wald *χ*^*2*^ = 9.62, *p* = 0.002, *OR* = 2.36 (95% = [1.37–4.06]), controlling for participants’ current age, ethnicity, gross annual income in their current household, and highest level of completed education. A complete overview of self-reported perceived misdiagnoses is presented in Table 3.

**Table 3:** Observed rates of perceived misdiagnoses for specific psychiatric conditions and logistic regression parameters.

	Perceived Misdiagnoses								
	Male		Female		Total		Logistic Regression		
	N	%	N	%	N	%	*p*	*OR*	95% CI
Personality disorders	35	6.1	114	17.9	149	12.3	<0.001	3.50	2.35–5.23
Anxiety disorders	25	4.4	55	8.6	80	6.6	0.002	2.22	1.36–3.64
Mood disorders	22	3.8	53	8.3	75	6.2	<0.001	2.57	1.52–4.32
Chronic fatigue syndrome/burnout-related disorders	21	3.7	28	4.4	49	4	0.296	1.41	0.74–2.71
Attention-deficit/hyperactivity disorder	18	3.1	16	2.5	34	2.8	0.500	0.79	0.40–1.57
Post-traumatic stress disorder/trauma-related disorders	3	0.5	13	2.0	16	1.3	–	–	–
Fibromyalgia	2	0.3	6	0.9	8	0.7	–	–	–
Eating disorders	0	0.0	2	0.3	2	0.2	–	–	–
Substance use disorders	1	0.2	0	0.0	1	0.1	–	–	–
Oppositional defiant disorder/conduct disorder	0	0.0	1	0.2	1	0.1	–	–	–

*Note*. The % values under the column Total reflect the rate of participants who reported a misdiagnosis for each psychiatric condition relative to the entire sample of adult participants (*N* = 1211). The % values under the Male and Female columns reflect the rates of men and women who reported a misdiagnosis relative to the entire sample of men and women, respectively. Some logistic regressions were not performed due to insufficient sample size. *OR*, Odds Ratio.

The average number of perceived misdiagnoses was 0.38 (*SD* = 0.80, range: 0–7), with 15% of the sample reporting a misdiagnosis of only one psychiatric condition, 6.4% reporting two perceived misdiagnoses, while an additional 2.5% reported three or more perceived misdiagnoses. Autistic women reported significantly more perceived misdiagnoses (*M* = 0.50, *SD* = 0.89, range: 0–7) compared to men (*M* = 0.25, *SD* = 0.66, range: 0–6; *t* (1209) = 5.59, *p* < 0.001, 95% CI of the difference = [0.17–0.34]). A complete overview of the proportion of participants who reported a certain number of perceived misdiagnoses for prior psychiatric conditions is presented in Table 4.

**Table 4:** Number of perceived misdiagnoses.

Count	Perceived misdiagnoses					
	Male		Female		Total	
	N	%	N	%	N	%
0	478	83.3	435	68.3	913	75.4
1	63	11.0	119	18.7	182	15
2	22	3.8	56	8.8	78	6.4
3	9	1.6	21	3.3	30	2.5
4	1	0.2	5	0.8	6	0.5
6	1	0.2	0	0	1	0.1
7	0	0	1	0.2	1	0.1
**Total**	574	100	637	100	1211	100

### Perceived misdiagnoses as a function of age of autism diagnosis

Finally, age of autism diagnosis was categorized into different groups, in order to explore whether individuals who were diagnosed with autism within a certain age range were more or less likely to report perceived misdiagnoses, and whether different patterns of perceived misdiagnoses for specific diagnostic categories were present for certain age groups. Out of the total sample, 24.8% (*n* = 274) of participants were diagnosed with autism below the age of 18, 9.8% (*n* = 108) were diagnosed between the ages of 18–24 years, 16.9% (*n* = 187) were diagnosed between 25 and 34 years, 21.2 (*n* = 234) were diagnosed between 35 and 44 years, 18.3% (*n* = 202) were diagnosed between 45 and 54 years, while the remaining 9% (*n* = 99) were diagnosed at or over the age of 55. A chi-square test of independence was performed in order to examine the association between age of autism diagnosis category and reporting at least one perceived misdiagnosis of a prior psychiatric condition. Participants who were diagnosed with autism between the ages of 18–54 years were more likely to report at least one perceived misdiagnosis relative to participants who were diagnosed with autism before the age of 18 and at or after the age of 55, χ2 (5, 1104) = 93.13, *p* < 0.001. A complete overview of the proportion of participants in each age category who reported at least one perceived misdiagnosis is presented in Table 5. Table 6 presents the frequencies of perceived misdiagnoses of specific psychiatric conditions as a function of age of autism diagnosis.

**Table 5:** Frequencies of at least one prior psychiatric diagnosis and at least one perceived misdiagnosis as a function of age of autism diagnosis.

	At least one prior psychiatric diagnosis (*n* = 1104)				At least one perceived misdiagnosis (*n* = 481)			
	Yes		No		Yes		No	
	*n*	%	*n*	%	*n*	%	*n*	%
<18	46	16.8	228	83.2	17	37.0	29	63.0
18–24	65	60.2	43	39.8	35	53.8	30	46.2
25–34	110	58.8	77	41.2	76	69.1	34	30.9
35–44	117	50.0	117	50.0	77	65.8	40	34.2
45–54	105	52.0	97	48.0	58	55.2	47	44.8
≥55	38	38.4	61	61.6	15	39.5	23	60.5
**Total**	481	100	623	100	278	100	203	100

**Table 6:** Frequencies of perceived misdiagnoses of specific psychiatric conditions as a function of age of autism diagnosis.

	Age of autism diagnosis											
	<18		18–24		25–34		35–44		45–54		≥55	
	*n*	%	*n*	%	*n*	%	*n*	%	*n*	%	*n*	%
Personality disorders	2	0.7	21	19.4	46	24.6	44	18.8	24	11.9	3	3
Anxiety disorders	2	0.7	8	7.4	26	13.9	16	6.8	17	8.4	7	7.1
Mood disorders	0	0	10	9.3	14	7.5	18	7.7	17	8.4	11	11.1
Chronic fatigue syndrome/burnout-related disorders	1	0.4	3	3.8	9	4.8	18	7.7	12	6.0	5	5.1
Attention-deficit/hyperactivity disorder	9	3.3	4	3.7	6	3.2	5	2.1	7	3.5	1	1
Post-traumatic stress disorder/trauma-related disorders	0	0	1	0.9	4	2.1	5	2.1	5	2.5	0	0
Fibromyalgia	0	0	0	0	3	1.6	1	0.4	2	1	1	1
Eating disorders	0	0	0	0	2	1.1	0	0	0	0	0	0
Substance use disorders	0	0	0	0	0	0	0	0	1	0.5	0	0
Oppositional defiant disorder/conduct disorder	0	0	0	0	1	0.5	0	0	0	0	0	0

## Discussion

Within a sample of 1211 primarily late-diagnosed autistic men and women, almost a quarter reported a perceived misdiagnosis of a psychiatric condition diagnosed prior to their autism diagnosis. Women (31.7%) reported perceived misdiagnoses more frequently than men (16.7%), and were significantly more likely to report perceived misdiagnoses of personality disorders, anxiety disorders, and/or mood disorders. However, women (65.8%) in general also reported prior diagnoses more frequently than men (34.2%), and within the group with prior diagnoses, no gender differences were observed. Although these results constitute a first step towards demonstrating that autistic adults, and particularly women, may be at greater risk of experiencing misdiagnoses for psychiatric conditions before obtaining a diagnosis of autism, we encourage future studies based on larger, more representative samples of autistic adults to replicate current findings.

In line with current findings, previous research examining perceived misdiagnoses in a sample of adults who identified as either autistic, suspected autistic, or non-autistic, showed that a combined 42% of autistic participants and 54% of possibly autistic participants either disagreed or only partially agreed with their mental health diagnosis, as opposed to only 14% of non-autistic participants.^5^ Recent meta-analytic work has documented that the prevalence of co-occurring conditions in autistic adults is between 55 and 57%, and that most psychiatric conditions show significantly higher prevalence rates in autistic adults compared with adults from the general population.^3^^,^^15^^,^^50^ This suggests that autistic adults are more likely to receive psychiatric diagnoses compared with non-autistic adults,^5^^,^^50^ but less likely to agree with these diagnoses.^5^ Only one previous study^29^ reported the frequency of misdiagnoses of specific psychiatric conditions in 136 adults, of whom only 58 had a formal autism diagnosis, identifying anxiety disorders, depressive disorders, other developmental disorders, and personality disorders as the psychiatric conditions with the highest rates of misdiagnosis. Our study, in a larger sample of autistic adults, adds to these findings, suggesting that personality, anxiety and mood disorders are likely misdiagnoses in autistic adults, and in autistic women in particular.

Perceived misdiagnoses may reflect genuine cases of misdiagnosed psychiatric conditions. Previous studies have documented overlapping symptoms between autism and several psychiatric conditions.^22^, ^23^, ^24^^,^^26^^,^^27^^,^^51^ For example, some of the strategies used by autistic adults to minimise social difficulties may be misconstrued as symptoms of social anxiety,^2^^,^^52^ whereas overlapping features between autism and ADHD in domains such as social functioning^19^^,^^20^ could allow autistic traits to be misinterpreted as symptoms of inattention and/or hyperactivity.^51^^,^^53^^,^^54^ There is also increasing clinical recognition that recurrent and/or treatment-resistant depression may potentially reflect an underlying neurodevelopmental condition, such as autism, missed due to overlapping clinical symptoms (e.g., social withdrawal).^55^ Lack of awareness of the presentation of autism in adulthood may also contribute to misdiagnoses of psychiatric conditions in adults with undiagnosed autism.^5^^,^^28^^,^^39^^,^^56^ For example, late-diagnosed autistic adults often report being viewed by healthcare professionals as high-functioning and perceived as coping during diagnostic assessments,^5^^,^^18^^,^^32^ despite facing significant challenges in daily life.^6^^,^^29^^,^^57^ This raises the likelihood that neurodevelopmental diagnoses will be ruled out and autistic traits misattributed to a different psychiatric diagnosis.^11^^,^^18^^,^^53^ Finally, camouflaging autistic traits may also hinder the accurate identification of autism in adults, particularly in autistic women.^58^ These processes may produce a mismatch between underlying autistic characteristics and external observable behaviour and therefore mask autistic traits during the diagnostic process.^2^^,^^29^^,^^59^ Although camouflaging and compensation facilitate adaptation into the neurotypical world^34^, ^35^, ^36^ and allow autistic individuals to feel safe,^60^ they may also result in delayed diagnosis of autism until late adolescence or even adulthood, and partly explain the gender differences in misdiagnoses reported in our study.^5^^,^^34^, ^35^, ^36^

An alternative explanation is that individuals later diagnosed as autistic may have initially been diagnosed with psychiatric conditions that were more readily observable at the surface level, but the underlying difficulty, autism, was missed. Patterns of co-occurrence between autism and mental health conditions likely originate from various sources, including shared underlying neural, affective, and cognitive mechanisms pervading autism and mental health conditions,^61^ or accumulated adverse life experiences associated with being autistic.^15^^,^^61^^,^^62^ One the one hand, prior diagnoses could reflect distinct co-occurring conditions, with symptom presentation identical to what would be observed in non-autistic individuals.^63^^,^^64^ On the other hand, prior diagnoses could reflect mental health difficulties that resulted from living without an established autism diagnosis and with limited access to appropriate care and support.^2^^,^^6^^,^^11^^,^^65^ Indeed, autistic adults who express disagreement with their co-occurring psychiatric diagnoses often report the perception that their psychiatric symptoms resulted from the challenges of living with undiagnosed autism rather than distinct co-occurring conditions.^5^^,^^59^ Autistic youths and adults may experience discrimination, loneliness, bullying, social exclusion, difficulty accessing employment or healthcare, as well as other adverse interpersonal events that can result in long-term trauma, symptoms of post-traumatic stress, difficulties navigating social situations, and unmet needs for social engagement.^61^, ^62^, ^63^ These adverse interpersonal events can also prompt a negative feedback loop, wherein autistic adults face negative interpersonal experiences, grow less motivated to seek social contact, and become increasingly isolated, lonely, or anxious.^61^ Moreover, previous quantitative and qualitative studies focusing on late-diagnosed autistic adults have documented poor past experiences with mental health professionals, prolonged mental health treatment before obtaining access to an autism assessment, lack of clinician awareness regarding the presentation of autism in adulthood, as well as multiple or inappropriate referrals.^8^^,^^10^^,^^32^^,^^65^, ^66^, ^67^, ^68^ Such experiences were often accompanied by long gaps between first concern, professional assessment, and diagnosis.^11^^,^^37^^,^^67^^,^^69^ Delays in obtaining an autism diagnosis and accessing appropriate support may also lead to problems such as social isolation, interpersonal friction, confusion, guilt, depression, or anxiety, which may in turn culminate in different mental health difficulties.^5^^,^^6^^,^^66^^,^^68^^,^^70^ Diagnoses resulting from mental health symptoms that are secondary to the key characteristics of autism may be perceived as misdiagnoses by autistic adults, even though they meet formal criteria for a co-occurring diagnosis. The complex relationship between autism and mental health conditions necessitates the development of mental health assessments capable of disentangling whether functional impairment can be attributed to co-occurring psychiatric symptoms and therefore warrants a diagnosis of a co-occurring mental health condition in the autistic adult, or to key characteristics of autism and therefore does not warrant a distinct diagnosis of a co-occurring mental health condition.^15^ Providing timely diagnosis and support is crucial in order to promote autistic flourishing, health, and wellbeing.

Our finding of one in four autistic adults, and one in three autistic women, reporting a perceived misdiagnosis is alarming and has clear implications for clinical practice. In this largest quantitative exploration of perceived misdiagnoses to date, we confirm existing qualitative accounts on misdiagnosed psychiatric conditions in adults with (undiagnosed) autism.^5^^,^^6^^,^^10^^,^^11^^,^^28^ Previous studies have linked misdiagnoses to longer diagnostic pathways, delayed recognition of autism, distrust towards healthcare professionals, and mental health problems such as depression or anxiety.^10^^,^^11^^,^^28^^,^^30^^,^^57^^,^^68^ Clinicians working with adults, both men and women, are therefore encouraged to remain alert to the nuanced presentations of autistic traits and consider the impact of compensatory and camouflaging strategies on the behavioural presentation of autism. Importantly, clinicians should be aware that autistic men and women are potentially susceptible to different types of misdiagnoses, with women in particular being more susceptible than men to misdiagnosis of personality disorder.

The present findings should be considered in light of some important limitations. First, information regarding participants' autism diagnoses was based on self-report data. The current study included only participants with an established diagnosis of autism obtained through qualified clinicians unaffiliated with the current study, and participants who were unsure about the status of their autism diagnosis were excluded from the analysis. Although self-reported diagnoses can be a reliable source,^71^ it is important to highlight that, in the current study, it was not possible to independently validate autism diagnoses through clinical reports or medical records. However, healthcare professionals' perspectives can also be prone to subjectivity, further highlighting the need to develop objective tools in (adult) autism assessment.^58^ Second, information regarding participants' perceived misdiagnoses was also based on self-report data. Participants were asked to indicate whether they believed that any of their earlier diagnoses outside of the autism spectrum were correct, and, if their response to the initial question was positive, selected one or more options from a list of diagnostic categories. Therefore, it was not possible to validate participants' self-reported perceived misdiagnoses, or distinguish between those who had experienced genuine misdiagnoses and those who disagreed with one or more of their prior diagnoses because they viewed them as by-products of living with undiagnosed autism. The clear wording of the question used to assess perceived misdiagnoses likely circumvented this limitation to a large extent. Current findings are also restricted to broader categories of psychiatric conditions (e.g., personality disorders, anxiety disorders) and therefore cannot be used to distinguish whether specific psychiatric conditions within each category are more likely than others to be perceived as misdiagnoses. Third, a substantial proportion of respondents had a high education level and did not have intellectual disabilities, which likely limits the current sample's representativeness of individuals across the full autism spectrum. The use of a self-report format may have excluded individuals with language or communication difficulties, raising the possibility that current findings may not be generalisable across the autism spectrum, but rather primarily reflect the experiences of individuals with no language or communication difficulties, no intellectual disabilities, and relatively high education level. Fourth, sampling bias could potentially limit the generalizability of conclusions drawn from the current study. Self-selection bias could be present, as the NAR only represents approximately 1% of the Dutch autistic population and relies on a convenience sample by recruiting participants on an entirely free and voluntary basis. The longitudinal nature of the NAR may also lend itself to non-response bias. Data analysed in the current study were drawn from the fifth annual data collection wave of the NAR. Individuals who had already participated in one or more previous waves received an invitation to complete the fifth wave, with 38.6% not returning to the survey. Comparing individuals who participated in the fifth wave and those who did not across multiple characteristics only showed that returning participants were on average 4.2 years older. Moreover, efforts to reach new participants are continuous, as the survey is regularly advertised nationwide in conferences, public talks, press articles, or presentations given by NAR researchers. We believe that most participants interested in joining the survey, including those who may be harder to reach, have the opportunity to learn about it, mitigating the presence of non-response bias. Nevertheless, we cannot fully rule out the presence of non-response bias, and suggest that results from the current study are interpreted with this limitation in mind. Fifth, the current study only assessed perceived misdiagnoses of prior psychiatric conditions in individuals who have already received a formal diagnosis of autism. However, there is a proportion of individuals who may qualify for an autism diagnosis but have not yet formally received one, and the current study cannot assess perceived misdiagnoses in this group of individuals. Finally, the current study did not include a control group. We therefore highlight the need for additional research comparing rates of perceived misdiagnoses in autistic and non-autistic individuals.

This study highlighted that a large proportion of autistic adults perceived that they had been misdiagnosed with one or more psychiatric conditions that were diagnosed sometime prior to receiving an autism diagnosis. Autistic women were more likely than men to report one or more perceived misdiagnoses, and differed with regard to the specific psychiatric conditions they were more likely to be misdiagnosed with. These findings constitute a first step towards demonstrating that autistic adults, and particularly women, may be more likely to experience misdiagnosis of one or more psychiatric conditions before eventually obtaining a formal autism diagnosis, thus highlighting the need for healthcare professionals to remain aware of the presentation of autism in adulthood and screen for autism earlier in adults presenting with numerous prior or existing diagnoses, such as personality, anxiety, or mood disorders. Mental health assessments should form an integral component of clinical care with regular screening, evaluation, and treatment done as part of ongoing support for autistic adults, rather than treating psychiatric conditions in isolation. Given then complex relationship between autism and psychiatric conditions in adulthood, additional epidemiological studies based on larger and more representative samples of autistic adults are needed to replicate current findings, and provide robust estimates of the overall frequency of misdiagnoses as well as the frequency of misdiagnoses for specific psychiatric conditions.

## Contributors

VK: Conceptualization, Data Curation, Formal Analysis, Investigation, Methodology, Validation, Visualization, Writing—original draft, Writing—review & editing. LL: Methodology, Validation, Writing—review & editing. RG: Methodology, Validation, Writing—review & editing. RH: Methodology, Validation, Writing—review & editing. SB: Conceptualization, Formal Analysis, Funding Acquisition, Investigation, Methodology, Project Administration, Supervision, Validation, Visualization, Writing—review & editing. VK, RG, and SB accessed and verified all of the study data. All authors read and approved the final version of the manuscript.

## Data sharing statement

Data collected for the study can be made available upon request. Information that will be made available includes deidentified participant data, data dictionary, plan of analysis, and analysis script. Requested data will be shared following a signed data access agreement. Data requests can be made by contacting the corresponding author (v.k.kentrou@vu.nl).

## Declaration of interests

The authors declare no competing interests.
